# Retrospective Reports of Parental Feeding Practices and Current Eating Styles in Polish Adults

**DOI:** 10.3390/nu15194217

**Published:** 2023-09-29

**Authors:** Aleksandra Małachowska, Marzena Jeżewska-Zychowicz

**Affiliations:** Department of Food Market and Consumer Research, Institute of Human Nutrition Sciences, Warsaw University of Life Sciences (SGGW-WULS), Nowoursynowska 159C, 02-776 Warsaw, Poland; marzena_jezewska_zychowicz@sggw.edu.pl

**Keywords:** parental feeding practices, childhood food experiences, eating style, intuitive eating, external eating, restrained eating

## Abstract

The role of childhood food experiences (CFEs) in determining future eating behaviors remains unclear. The aim of the study was to examine the link between CFEs and selected eating styles (ESs), i.e., intuitive (IE), restrained (ResEat) and external (ExtEat) eating, among 708 Polish adults aged 18–65 (477 women and 231 men). CFEs were measured with the Adults’ Memories of Feeding in Childhood questionnaire. Polish versions of the Intuitive Eating Scale-2 and Dutch Eating Behavior Questionnaire were used to assess ESs. Mann-Whitney U test was used to compare ESs scores between those with lower and higher CFEs. In the total sample, “Restrictions” and “Pressure and Food Reward” parental feeding practices favored lower IE, while “Healthy Eating Guidance” and “Monitoring” practices predisposed higher levels of IE in adulthood. “Restrictions” were found to correlate with greater chances of ResEat, whereas “Healthy Eating Guidance” was linked with lower probability of ResEat. “Pressure and Food Reward” and “Monitoring” were associated with higher score for ExtEat. “Restrictions”, “Child Control”, “Monitoring” and “Healthy Eating Guidance” practices were differently linked to ESs in women and men. The findings suggest that education programs for parents should focus on the long-term consequences of feeding practices.

## 1. Introduction

Childhood food experiences (CFEs) stem from diverse parental feeding practices (PFPs), such as pressuring to eat, teaching about nutrition, applying restrictions, monitoring a child’s food intake or using food as a reward [1,2]. PFPs can greatly influence a child’s eating, predisposing to favorable or unfavorable behaviors [3]. There is an ongoing debate on whether dietary behaviors established during early childhood (birth to 6 years) and middle childhood (6 to 12 years) continue into adolescence and further into adulthood [2,4]. A few longitudinal studies confirmed the moderate transmission of childhood dietary patterns into young adulthood [5,6,7]. Nevertheless, results from retrospective studies in adults indicated that selected CFEs may favor future eating behaviors especially in the form of eating styles (ESs) [8,9,10,11,12,13,14,15,16]. ESs describe psychological aspects of dietary behaviors, such as beliefs, attitudes, motives and feelings towards food and eating [17]. The role of selected ESs, e.g., intuitive eating (IE), restrained eating (ResEat) and external eating (ExtEat), as predictors of the food intake was confirmed in available research [17,18,19,20]. IE is a non-dieting approach that promotes eating based on physiological internal signals rather than emotional (EmoEat) or external (ExtEat) ones [21]. Identification of the individual’s ES and searching for its origin may be useful in prevention and treatment of diet-related diseases [22].

To date, research has confirmed the following associations: (1) the link between parents using food to control the child’s behavior (e.g., food pressuring or food rewarding) and more frequent binge-eating or dietary restraint [8], long-lasting food rejection [13], lower level of intuitive eating and higher level of disordered eating in adulthood [9]; (2) the association between parents applying restrictions or emotional regulation feeding and food preoccupation as well as emotional eating in adulthood [10,12]; (3) the link between positive and negative memories about family’s food rules and dieting in adulthood [14]; and (4) the association between having regular family meals as a child and having regular family meals as an adult [15,23]. Moreover, the availability of unhealthy foods at home during childhood was associated with less healthful eating [24] and early life eating environments with the development of the food addiction [25] in young adults.

Despite some evidence that there is a link between CFEs and maladaptive ESs in adulthood, e.g., ResEat or ExtEat, the existing studies have mostly included single or selected PFPs [8,9,10,11,12,13,14,15,16]. Moreover, the majority of research focused on the PFPs with potential short- and long-term negative impacts, such as food rewarding, pressuring to eat, rigid controlling behaviors, etc. [8,9,10,11,12,13,14,15]. Little is known about the link between diverse CFEs and adaptive ESs in adulthood, such as IE [26]. Adaptive ESs are believed to be characterized by the absence or low levels of maladaptive eating, hence they may be differently related to CFEs [21]. Furthermore, few available studies have indicated that PFPs may be gendered, and thus have different impacts on girls’ and boys’ eating [27,28,29]. Therefore, it may be hypothesized that CFEs related to the same PFPs can differently predict ESs among women and men [11,26]. 

The aim of the study was to examine the association between childhood food experiences and selected eating styles separately in adult women and men. The following hypotheses were tested: 1. Childhood food experiences such as healthy eating guidance, monitoring and child control predispose to a greater level of intuitive eating in adulthood, while experiences related to restrictions, food rewarding or pressuring to eat favor restrained and external eating in adulthood; 2. Gender differentiates the association between childhood food experiences and selected eating styles in adulthood.

## 2. Materials and Methods

### 2.1. Study Design and Participants

The data for this cross-sectional study were collected in Poland from October 2022 to January 2023 with the use of a Computer-Assisted Web Interview technique. An invitation to participate in the study with a short description of its purpose and a link to a questionnaire were published on social media. The inclusion criteria were age between 18 and 65, ability to read and understand Polish and informed online consent. The exclusion criteria were as follows: age lower than 18 years old or greater than 65 years old, inability to read and understand Polish and lack of consent. The data anonymity and confidentiality were assured. The final study sample included 477 women and 231 men (708 participants in total), who properly filled out the questionnaire. The study design was approved by the Ethics Committee of the Institute of Human Nutrition Sciences, Warsaw University of Life Sciences, in Poland (Resolution No. 02/2020) and was conducted in compliance with the Declaration of Helsinki.

### 2.2. Measures

#### 2.2.1. Assessment of the Childhood Food Experiences

Adults’ memories of childhood food experiences related to PFPs were assessed with The Adults’ Memories of Feeding in Childhood questionnaire (AMoFiC) [2]. The tool had a 5-factor structure: 1. “Restrictions” (13 items), 2. “Healthy Eating Guidance” (9 items), 3. “Pressure and Food Reward” (6 items), 4. “Monitoring” (5 items), 5. “Child Control” (6 items). The items were rated with a 5-point Likert scale, ranging from never/disagree (1) to always/agree (5). The additional answer, i.e., “I don’t remember”, was scored zero points and was further treated as a missing value. The score for each subscale was obtained by calculating mean values.

#### 2.2.2. Assessment of the Eating Styles

Intuitive eating (IE) was assessed with the Polish version of the Intuitive Eating Scale-2 [30]. It had a 4-factor structure: 1. “Reliance on Hunger and Satiety Cues” (6 items), 2. “Eating For Physical Rather Than Emotional Reasons” (4 items), 3. “Body–Food Choice Congruence” (3 items), 4. “Unconditional Permission to Eat” (3 items). The items were rated with a 5-point Likert scale, ranging from strongly disagree (1) to strongly agree (5). 

Restrained and external eating were assessed with the Polish version of the Dutch Eating Questionnaire [17]. “Restrained eating” (ResEat) subscale contains 9 items, whereas “External eating” (ExtEat) is a 7-item subscale. The items were rated with a 5-point Likert scale, ranging from never (1) to very often (5). Scores for each IE subscale as well as for ResEat and ExtEat were presented as mean values.

#### 2.2.3. Sociodemographic Characteristics

The following sociodemographic data were collected: age, gender (female or male), education (primary, lower secondary, upper secondary, or higher) and place of residence (village, town below 20,000 inhabitants, town between 20,000 and 100,000 inhabitants, or city with over 100,000 inhabitants).

### 2.3. Data Analysis

Sociodemographic characteristics were presented with the descriptive statistics. Two groups of participants were distinguished based on their scores for each subscale from the AMoFiC questionnaire: 1. “lower childhood food experiences (lower CFEs)”—mean score below or equal to 3.00, and 2. “higher childhood food experiences (higher CFEs)”—mean score greater than 3.00. Shapiro–Wilk test was used to test the normality of distribution. Mean scores for the four subscales from the Intuitive Eating Scale-2 as well as for Restrained and External eating subscales were compared between those two groups, i.e., lower and higher CFEs, with the Mann–Whitney U test. Significance level was set at α = 0.05. All analyses were performed in IBM SPSS Statistics for Windows, version 28.0 (IBM Corp., Armonk, NY, USA).

## 3. Results

### 3.1. Participants

The study included a total of 708 participants. The majority of the study group were women (67.4%), people with higher education (83.2%) and people living in a city (62.6%) (Table 1). The mean age of the participants was 36.9 ± 11.5 years.

### 3.2. Childhood Food Experiences and Intuitive Eating in Adulthood

Participants with lower CFEs related to “Restrictions” had higher scores in three subscales of IE, i.e., “Reliance on Hunger and Satiety Cues”, “Eating For Physical Rather Than Emotional Reasons” and “Unconditional Permission to Eat”, in comparison to people with higher experiences, which was confirmed in the total sample and among women (Table 2). Among men, those with lower experiences of “Restrictions” scored higher in “Unconditional Permission to Eat” and lower in “Body–Food Choice Congruence”. CFEs related to “Healthy Eating Guidance” were linked to higher scores for “Reliance on Hunger and Satiety Cues”, “Eating For Physical Rather Than Emotional Reasons” and “Body–Food Choice Congruence” in the total sample as well as among women and men separately. The experience of “Pressure and Food Reward” in childhood was associated with a lower score for “Eating For Physical Rather Than Emotional Reasons” in the total sample and in women and men separately. Higher experience of “Monitoring” of the child’s eating behaviors favored higher score in “Body–Food Choice Congruence” in the total sample, as well as in women. The experiences of “Child Control” differentiated the score of “Eating For Physical Rather Than Emotional Reasons” in men only, with the greater score among the group with higher experiences of this PFP (Table 2).

### 3.3. Childhood Food Experiences, Restrained and External Eating in Adulthood

The differences in ResEat were noted only in regard to the “Restrictions” and “Healthy Eating Guidance” subscales (Table 3). Participants who had more experiences of “Restrictions” obtained a higher score for ResEat in comparison to those with lower experiences in the total sample and separately among women and men. Higher experiences of the PFPs related to “Healthy Eating Guidance” were linked to a lower score for ResEat in the total sample, yet no significant differences were noted within the women and within the men. Higher childhood experiences of “Pressure and Food Reward” as well as “Monitoring” were associated with a higher score for ExtEat in the total sample. In the female and male group, only differences resulting from the experiences of the “Pressure and Food Reward” were observed. Both men and women with higher experiences of this PFP obtained a greater score for ExtEat in comparison to the group with lower experiences (Table 3).

## 4. Discussion

The reported differences in the scores for certain eating styles (ESs), i.e., intuitive (IE), restrained (ResEat) and external (ExtEat) eating, in regard to the declared childhood food experiences (CFEs) have confirmed the link between parental feeding practices (PFPs) and dietary behaviors in adulthood. Nevertheless, some results underlined the complexity of this relationship, for example individual IE components were differently associated with CFEs. Moreover, different CFEs explained ResEat and ExtEat in adulthood.

The study results indicate that restrictions in childhood can be linked to lower IE in adulthood. Restrictions, specifically for weight control, may therefore contribute to relying on internal signals less often, eating for reasons unrelated to physical hunger and not allowing oneself to eat whatever is desired [30]. This particular PFP was the least reported one in the study sample; however, it was more common in women. The CFEs of restrictions in women seem to differently modify their ability of IE in comparison to men. Previous research suggests that maternal ESs may differently predict child’s ESs, especially in regard to restrained eating [28]. Maternal ResEat may predict daughters’ ResEat only [28]. This can be one of the factors explaining different results in women and men in the current study. The sociocultural models that indicate that dietary restrictions are more specific for girls and women in comparison to boys and men [31] seem to explain our findings. We also observed that the results for certain IE components did not differ between men with lower and higher experiences of restrictions in childhood. Moreover, a higher tendency for making food choices that promote better body functioning was observed only in men who have experienced restrictions during childhood. Nevertheless, the role of the childhood restrictions in determining dietary behaviors in men should be further investigated [11,26].

The experiences of parental guidance on healthy eating in childhood favored greater compliance with IE in the total sample as well as in women and men separately, except for giving oneself permission to eat desired foods. Therefore PFPs such as encouraging children in a supportive way to try new foods and eat diversely, modelling healthy eating, involving children in meal planning and preparations seem to favor eating in response to internal cues and possibly promote more healthful diets [2,32,33]. As parental influence is the first factor that may affect a child’s eating, the abovementioned practices can promote the inborn ability of IE and prevent the child from eating disturbances later in life [3,34]. 

The experiences of being pressured to eat or being rewarded with food were linked to more common use of food to deal with unpleasant emotions in the total sample and in both women and men. Such experiences may consequently lead to emotional eating and overeating [28]. This result is contrary to the previous ones that suggested different brain responses to rewarding in women and men [35] and among boys and girls [29,36,37]. It may in turn differently determine diet quality in female and male adults [4]. Perhaps the difference between women’s and men’s brain processing might be more significant in terms of determining ESs in childhood [29,36,37] rather than in adulthood.

Being monitored by parents in terms of types of foods eaten during childhood favored choosing foods that promote better body functioning in adulthood, in the total sample and in women. Parental control, i.e., being responsive to child’s needs and wants, was linked to eating only for physical reasons in men. Previous research has shown that the role of both of these PFPs in determining dietary behaviors in adulthood remains unclear and possibly less significant in comparison to other parenting practices [2,4]. The differences between women and men might result from varying maternal and paternal influences or from different impacts of other caregivers [27,28]; however, it was not measured in this study. Paying attention to the type of foods eaten by the child and being responsive to the child’s needs may support healthful long-term outcomes [2,3].

Studies previously conducted on IE have shown several differences between women and men, similarly as this study. The total Intuitive Eating Scale-2 score and subscales for women were significantly lower, except for the one related to the ability of relying on internal signals [38,39]. Women may be more attentive to body signals than men [40]. However, the focus on appearance, weight control behaviors and disordered eating are observed among males as well [41]. A recent meta-analysis found that across a variety of age groups and cultures IE is higher in men in comparison to women [42,43]. This may be the result of an increased societal pressure that women face in terms of body dissatisfaction and internalization of a thin ideal [44]. Evidence suggests that men might also face pressure to conform to ideals [45], yet the one women face is more frequent and severe [44]. Another explanation for the differences in IE scores between men and women may relate to the relationship between personality and IE scores. For example, men with greater global self-esteem (GSE), thus also with narcissistic features, are less likely to give themselves permission to eat what is desired [46]. On the other hand, in women, greater GSE might promote IE [46]. The lower IE in women may also stem from the link between parental warmth and overprotection, which might cause greater parental concern, and in turn decrease eating in response to internal signals [26]. The assumption that different dietary experiences may correlate differently with IE scores in women and men was confirmed in the study group.

Contrary to the intergender differences related to the IE, having experienced restrictions in childhood predicted a greater ResEat score in both female and male adults as well as in the total sample. This association may result from the direct transmission of the behaviors learned during childhood or from the disturbances in the IE later in life [34]. Our observations suggest the complexity of ResEat [47] and the existence of different underlying mechanisms, such as non-food related adverse childhood experiences. For example, childhood abuse or neglect were found to predispose increased concerns about body shape and weight, unhealthy weight control behaviors, overeating or anxiety about food or eating [48,49]. The experience of financial deprivation in childhood was found to predict less favorable food choices in adulthood [50]. On the other hand, in the total sample, parental guidance on healthy eating predisposed to lower level of ResEat in adulthood. It may be concluded that this PFP may favor healthier eating behaviors, yet not in a form of strict, rigid food rules, but rather as a conscious choice, which is in line with the IE principles [19,30]. 

The practices such as pressuring to eat or food rewarding predisposed to greater ExtEat; however, differences between women and men were observed. Moreover, parental monitoring in terms of types of foods eaten favored greater ExtEat in the total sample, while solely in women parental guidance on healthy eating was linked to a lower ExtEat. Available studies on ExtEat are inconsistent and the role of ExtEat in promoting weight gain has been questioned [17,51,52,53]. Perhaps selected CFEs may mediate or moderate the relationship between ESs and dietary behaviors, thus further research should focus on explaining those results. 

The experiences of restrictions and parental control had no effect on ExtEat while pressuring and food rewarding, parental monitoring and parental control had no effect on ResEat. Thus, the hypotheses related to ResEat and ExtEat were only partially confirmed. On the other hand, hypotheses related to the IE were confirmed. It might be that CFEs are more useful for determining selected adaptive ESs (e.g., IE) rather than the nonadaptive ones (e.g., ResEat, ExtEat). Our findings confirmed a need to conduct more research that would examine the association between CFEs and adaptive ESs [26], also in a representative study sample. Additionally, CFEs seem to differentiate IE among women and men more than they differentiate ExtEat or ResEat, which supports our second hypothesis. The future studies on the link between CFEs and IE or other adaptive ESs, e.g., mindful eating, should include separate analysis of such associations in women and men to confirm these results. Moreover, although some significant results have been noted, differences in ESs between the groups with lower and higher CFEs were quite small. Thus, further research should be carried out to verify these observations. In addition, PFPs may be linked to parenting styles and other non-food related practices that may affect eating behaviors [54]. There is also a need for including those aspects in the longitudinal research for a better understanding of the development of adaptive and maladaptive eating behaviors [54].

The current study contributes to the growing body of literature on the development of eating styles in adulthood. Understanding which factors may contribute to the prevalence of different eating styles in adulthood is crucial, because they can be linked to both successful prevention of disordered eating behaviors and ensuring higher diet quality. The findings of this research indicate that the public health approaches should focus on seeking methods to improve CFEs, most notably by supporting the relationship between parents and children through nutrition education. The latter have the potential to reduce the development of various health-risk behaviors. Furthermore, investing in parenting interventions that target healthful family mealtime has the potential for a long-term impact on their own children’s parenting practices [55]. 

### Study Strengths and Limitations

This study is the first to assess the relationships between diverse childhood food experiences and selected eating styles in adulthood in the Polish adults. A relatively large study sample can be pointed out as another study strength. Nevertheless, the study also has a few limitations. Firstly, a cross-sectional character of the study does not allow us to draw any causal associations. Moreover, a non-representative study sample prohibits the generalization of the study results. The possible diverse impact of both parents, thus different use of PFPs and the impact of other caregivers, have not been included in this study. Additionally, only selected eating styles were chosen for this research. Lastly, the childhood food experiences were based on the retrospective reports, which might have been imprecise. Nevertheless, despite the fact that relying on the long-term memories may be subjected to bias, findings from the retrospective studies are worth considering. Even if the participants’ food memories are imprecise, the experiences from the past are meaningful and might underlie current beliefs, values or daily activities. Food- or eating-related memories can provide substantial information about people’s life and sociocultural environment [56,57], thus they can be used to explain behavior.

## 5. Conclusions

Findings from the current study suggest that childhood food experiences related to diverse parental feeding practices can differently determine selected eating styles, i.e., intuitive, external and restrained eating, in adulthood. Having experienced “Restrictions” in childhood favored lower intuitive eating (“Reliance on Hunger and Satiety Cues”, “Eating for Physical Rather Than Emotional Reasons”, “Unconditional Permission to Eat”) and higher restrained eating in adulthood. “Healthy Eating Guidance” predisposed to greater intuitive eating (“Eating for Physical Rather Than Emotional Reasons”, “Reliance on Hunger and Satiety Cues”, “Body-Food Choice Congruence”) and lower restrained eating in adulthood. “Pressure and Food Reward” correlated negatively with “Eating for Physical Rather Than Emotional Reasons” (intuitive eating) and positively with external eating. The experience of parental monitoring was linked to greater “Body-Food Choice Congruence” (intuitive eating) and external eating. “Restrictions”, “Healthy Eating Guidance”, “Monitoring” and “Child Control” were differently linked to intuitive eating components and external eating in women and men. The abovementioned findings suggest that parents’ education programs should focus on long-term consequences of feeding practices as well as the existence of the intergender differences among women and men, especially in regard to intuitive eating. Further retrospective studies in the representative samples are needed to confirm these findings. There is also a need for conducting longitudinal studies that would focus on the mechanism linking childhood food experiences and eating behaviors in adulthood.

## Figures and Tables

**Table 1:** Sociodemographic characteristics of the study sample.

Variables		Total (*n* = 708) *n* (%)	Women (*n* = 477) *n* (%)	Men (*n* = 231) *n* (%)
Age (years)	18–24	94 (13.3)	65 (13.6)	29 (12.5)
	25–39	349 (49.3)	253 (53.0)	96 (41.6)
	40–54	193 (27.2)	120 (25.2)	73 (31.6)
	55–65	72 (10.2)	39 (8.2)	33 (14.3)
Education	Primary	3 (0.4)	3 (0.6)	0 (0.0)
	Lower secondary	3 (0.4)	2 (0.4)	1 (0.4)
	Upper secondary	113 (16.0)	68 (14.3)	45 (19.5)
	Higher (e.g., BSc and MSc)	589 (83.2)	404 (84.7)	185 (80.1)
Place of Residence	Village	113 (15.9)	78 (16.3)	35 (15.2)
	Town below 20,000 inhabitants	43 (6.1)	31 (6.5)	12 (5.2)
	Town between 20,000 and 100,000 inhabitants	109 (15.4)	69 (14.5)	40 (17.3)
	City with over 100,000 inhabitants	443 (62.6)	299 (62.7)	144 (62.3)

*n*, number of participants; BSc, Bachelor of Science; MSc, Master of Science.

**Table 2:** Childhood food experiences and intuitive eating in the study sample.

Childhood Food Experiences (CFEs)			Intuitive Eating—Subscales			
Subscale	Groups of Respondents	*n* (%)	Reliance on Hunger and Satiety Cues Mean ± SD	Eating for Physical Rather than Emotional Reasons Mean ± SD	Body–Food Choice Congruence Mean ± SD	Unconditional Permission to Eat Mean ± SD
Total sample (*n* = 708)						
Restrictions	lower CFEs ^a^	666 (94.1)	3.42 ± 0.79 *	3.36 ± 1.15 **	3.42 ± 0.77	3.65 ± 0.81 **
	higher CFEs ^b^	42 (5.9)	3.07 ± 1.03 *	2.86 ± 1.10 **	3.60 ± 0.91	3.30 ± 0.81 **
Healthy Eating Guidance	lower CFEs	292 (41.2)	3.20 ± 0.85 ***	3.09 ± 1.19 ***	3.32 ± 0.77 ***	3.58 ± 0.86
	higher CFEs	416 (58.8)	3.53 ± 0.76 ***	3.50 ± 1.09 ***	3.51 ± 0.78 ***	3.66 ± 0.78
Pressure and Food Reward	lower CFEs	485 (68.5)	3.43 ± 0.81	3.45 ± 1.14 ***	3.46 ± 0.74	3.60 ± 0.82
	higher CFEs	223 (31.5)	3.33 ± 0.81	3.08 ± 1.14 ***	3.36 ± 0.85	3.67 ± 0.81
Monitoring	lower CFEs	479 (67.7)	3.38 ± 0.79	3.38 ± 1.14	3.38 ± 0.76 **	3.64 ± 0.79
	higher CFEs	229 (32.3)	3.44 ± 0.86	3.24 ± 1.16	3.53 ± 0.81 **	3.59 ± 0.87
Child Control	lower CFEs	538 (76.0)	3.43 ± 0.78	3.34 ± 1.13	3.45 ± 0.77	3.63 ± 0.80
	higher CFEs	170 (24.0)	3.30 ± 0.89	3.30 ± 1.22	3.38 ± 0.82	3.61 ± 0.86
Women (*n* = 477)						
Restrictions	lower CFEs	444 (93.1)	3.39 ± 0.83 *	3.16 ± 1.16 *	3.44 ± 0.80	3.62 ± 0.83 *
	higher CFEs	33 (6.9)	2.98 ± 1.05 *	2.73 ± 1.12 *	3.49 ± 0.89	3.34 ± 0.78 *
Healthy Eating Guidance	lower CFEs	196 (41.1)	3.15 ± 0.90 ***	2.85 ± 1.18 ***	3.35 ± 0.79 *	3.53 ± 0.89
	higher CFEs	281 (58.9)	3.51 ± 0.79 ***	3.32 ± 1.12 ***	3.51 ± 0.82 *	3.65 ± 0.78
Pressure and Food Reward	lower CFEs	316 (66.2)	3.40 ± 0.86	3.23 ± 1.15 **	3.48 ± 0.77	3.58 ± 0.84
	higher CFEs	161 (33.8)	3.30 ± 0.84	2.93 ± 1.16 **	3.37 ± 0.88	3.64 ± 0.80
Monitoring	lower CFEs	317 (66.5)	3.35 ± 0.82	3.16 ± 1.15	3.38 ± 0.78 **	3.64 ± 0.79
	higher CFEs	160 (33.5)	3.40 ± 0.93	3.06 ± 1.20	3.57 ± 0.85 **	3.54 ± 0.89
Child Control	lower CFEs	351 (73.6)	3.40 ± 0.83	3.16 ± 1.15	3.48 ± 0.80	3.61 ± 0.81
	higher CFEs	126 (26.4)	3.26 ± 0.92	3.05 ± 1.21	3.35 ± 0.82	3.57 ± 0.87
Men (*n* = 231)						
Restrictions	lower CFEs	222 (96.1)	3.46 ± 0.70	3.77 ± 0.99	3.39 ± 0.70 *	3.70 ± 0.78 *
	higher CFEs	9 (3.9)	3.41 ± 0.93	3.36 ± 0.94	3.96 ± 0.90 *	3.15 ± 0.91 *
Healthy Eating Guidance	lower CFEs	96 (41.6)	3.31 ± 0.73 **	3.56 ± 1.07 *	3.25 ± 0.72 *	3.68 ± 0.80
	higher CFEs	135 (58.4)	3.57 ± 0.68 **	3.88 ± 0.91 *	3.52 ± 0.69 *	3.67 ± 0.79
Pressure and Food Reward	lower CFEs	169 (73.2)	3.48 ± 0.71	3.85 ± 0.99 **	3.44 ± 0.69	3.64 ± 0.77
	higher CFEs	62 (26.8)	3.42 ± 0.71	3.48 ± 0.97 **	3.33 ± 0.76	3.76 ± 0.86
Monitoring	lower CFEs	162 (70.1)	3.43 ± 0.72	3.79 ± 1.01	3.40 ± 0.71	3.65 ± 0.79
	higher CFEs	69 (29.9)	3.54 ± 0.68	3.65 ± 0.96	3.43 ± 0.73	3.72 ± 0.79
Child Control	lower CFEs	187 (81.0)	3.48 ± 0.69	3.69 ± 1.00 *	3.40 ± 0.69	3.67 ± 0.78
	higher CFEs	44 (19.0)	3.41 ± 0.79	4.01 ± 0.95 *	3.46 ± 0.81	3.70 ± 0.83

*n*, number of participants; ^a^ mean score for the subscale below or equal to 3.00, ^b^ mean score for the subscale above 3.00; SD, standard deviation; significant at * *p* < 0.05, ** *p* < 0.01, *** *p* < 0.001; Mann–Whitney U Test.

**Table 3:** Childhood food experiences, external and restrained eating in the study sample.

Childhood Food Experiences (CFEs)			Eating Style	
**Subscale**	Groups of Respondents	*n* (%)	External Eating Mean ± SD	Restrained Eating Mean ± SD
Total sample (*n* = 708)				
Restrictions	lower CFEs ^a^	666 (94.1)	2.97 ± 0.71	2.58 ± 0.88 ***
	higher CFEs ^b^	42 (5.9)	3.08 ± 0.82	3.12 ± 0.82 ***
Healthy Eating Guidance	lower CFEs	292 (41.2)	3.05 ± 0.76	2.70 ± 0.90 *
	higher CFEs	416 (58.8)	2.92 ± 0.69	2.56 ± 0.87 *
Pressure and Food Reward	lower CFEs	485 (68.5)	2.89 ± 0.71 ***	2.60 ± 0.85
	higher CFEs	223 (31.5)	3.15 ± 0.71 ***	2.66 ± 0.95
Monitoring	lower CFEs	479 (67.7)	2.94 ± 0.73 *	2.58 ± 0.88
	higher CFEs	229 (32.3)	3.04 ± 0.69 *	2.69 ± 0.89
Child Control	lower CFEs	538 (76.0)	2.96 ± 0.69	2.60 ± 0.88
	higher CFEs	170 (24.0)	3.02 ± 0.79	2.67 ± 0.88
**Women (*n* = 477)**				
Restrictions	lower CFEs	444 (93.1)	3.02 ± 0.72	2.67 ± 0.89 **
	higher CFEs	33 (6.9)	3.18 ± 0.87	3.12 ± 0.74 **
Healthy Eating Guidance	lower CFEs	196 (41.1)	3.16 ± 0.78 **	2.80 ± 0.91
	higher CFEs	281 (58.9)	2.95 ± 0.69 **	2.63 ± 0.86
Pressure and Food Reward	lower CFEs	316 (66.2)	2.96 ± 0.72 ***	2.66 ± 0.85
	higher CFEs	161 (33.8)	3.18 ± 0.73 ***	2.77 ± 0.95
Monitoring	lower CFEs	317 (66.5)	3.02 ± 0.75	2.66 ± 0.88
	higher CFEs	160 (33.5)	3.07 ± 0.70	2.77 ± 0.88
Child Control	lower CFEs	351 (73.6)	3.00 ± 0.70	2.68 ± 0.89
	higher CFEs	126 (26.4)	3.13 ± 0.82	2.76 ± 0.87
**Men (*n* = 231)**				
Restrictions	lower CFEs	222 (96.1)	2.85 ± 0.68	2.42 ± 0.84 *
	higher CFEs	9 (3.9)	2.71 ± 0.47	3.16 ± 1.13 *
Healthy Eating Guidance	lower CFEs	96 (41.6)	2.82 ± 0.66	2.50 ± 0.86
	higher CFEs	135 (58.4)	2.86 ± 0.67	2.41 ± 0.86
Pressure and Food Reward	lower CFEs	169 (73.2)	2.77 ± 0.66 **	2.48 ± 0.85
	higher CFEs	62 (26.8)	3.05 ± 0.65 **	2.37 ± 0.88
Monitoring	lower CFEs	162 (70.1)	2.79 ± 0.66	2.43 ± 0.85
	higher CFEs	69 (29.9)	2.97 ± 0.69	2.50 ± 0.87
Child Control	lower CFEs	187 (81.0)	2.87 ± 0.68	2.45 ± 0.86
	higher CFEs	44 (19.0)	2.73 ± 0.61	2.43 ± 0.88

*n*, number of participants; ^a^ mean score for the subscale below or equal to 3.00, ^b^ mean score for the subscale above 3.00; SD, standard deviation; significant at * *p* < 0.05, ** *p* < 0.01, *** *p* < 0.001; Mann–Whitney U Test.

## Data Availability

The data presented in this study are available on request from the corresponding author.
